# Electrochemically Reduced Water Delays Mammary Tumors Growth in Mice and Inhibits Breast Cancer Cells Survival* In Vitro*

**DOI:** 10.1155/2018/4753507

**Published:** 2018-09-26

**Authors:** Giovanni Vanni Frajese, Monica Benvenuto, Rosanna Mattera, Saverio Giampaoli, Elena Ambrosin, Roberta Bernardini, Maria Gabriella Giganti, Loredana Albonici, Ivan Dus, Vittorio Manzari, Andrea Modesti, Maurizio Mattei, Roberto Bei

**Affiliations:** ^1^Department of Movement, Human and Health Sciences, University of Rome “Foro Italico”, 00135 Rome, Italy; ^2^Department of Clinical Sciences and Translational Medicine, University of Rome “Tor Vergata”, 00133 Rome, Italy; ^3^Department of Agriculture, Environment and Food, University of Molise, 86100 Campobasso, Italy; ^4^STA, University of Rome “Tor Vergata”, 00133 Rome, Italy; ^5^Nerò H2O d.o.o, Kraška Ulica 2, 6210 Sežana, Slovenia

## Abstract

Electrochemical reduced water (ERW) has been proposed to have beneficial effects on human health due to its rich content of H_2_ and the presence of platinum nanoparticles with antioxidant effects. Many studies have demonstrated that ERW scavenging properties are able to reduce the damage caused by oxidative stress in different experimental models. Although few* in vivo* studies have been reported, it has been demonstrated that ERW may display anticancer effects by induction of tumor cells apoptosis and reduction of both angiogenesis and inflammation. In this study, we show that ERW treatment of MCF-7, MDA-MB-453, and mouse (TUBO) breast cancer cells inhibited cell survival in a time-dependent fashion. ERW decreased ErbB2/*neu* expression and impaired pERK1/ERK2 and AKT phosphorylation in breast cancer cells. In addition, ERW treatment induced apoptosis of breast cancer cell lines independently of the status of p53 and ER and PR receptors. Our* in vivo* results showed that ERW treatment of transgenic BALB-*neu*T mice delayed the development of mammary tumors compared to the control. In addition, ERW induced a significant prolongation of tumor-free survival and a reduction in tumor multiplicity. Overall, these results suggest a potential beneficial role of ERW in inhibiting cancer cells growth.

## 1. Introduction

During electrolysis, water (H_2_O) weakly dissociates to H^+^ and OH^−^ in aqueous solution. The hydrogen ions, by receiving an electron on the cathode, are transformed in active and then molecular hydrogen (H_2_). Simultaneously, OH^−^ is oxidized to generate molecular oxygen O_2_. For these reasons, the water solution near the cathode is characterized by an alkaline pH, an elevated percentage of dissolved hydrogen and a small amount of platinum (Pt) nanoparticles: this solution is named electrochemical reduced water (ERW) [1]. Reduced water (electrochemical or natural) has been suggested to have beneficial effects on human health [1]. Reducing activity present in natural springs has been described to be beneficial for dermatological and antibacterial treatment [2]. It has been demonstrated that ERW shows reactive oxygen species- (ROS-) scavenging activity that could be useful in the treatment of oxidative stress-related diseases [3, 4]. The first studies to support the use of ERW were performed in Japan in the 1930s and in the 1960s and it was recognized that ERW was effective in chronic diarrhea, gastrointestinal fermentation, and gastric hyperacidity [1]. The antioxidant activity of ERW can be in part due to the extremely low oxidation reduction potential (ORP: average less than -400mV) and the presence of atomic hydrogen [5]. Another factor that could enhance the antioxidant effect of the ERW water is the presence of Pt nanoparticles produced by the electrolysis process in platinum-plated instruments. Pt nanoparticles have demonstrated to scavenge O_2_^−^, OH^•^, and H_2_O_2_ [6, 7], reducing the damage caused by oxidative stress in cell cultures [8]. In addition, platinum nanoparticles catalyze the process of converting the atomic hydrogen molecule to active atomic hydrogen [1]. It was reported that ERW inhibited the DNA breaking reaction in a dose-dependent manner [9]. ERW was also able to scavenge ROS in rat L6 myotube cells and to enhance sugar uptake [10]. In addition, ERW ameliorated the symptoms of diabetic mice [11–13]. The effects of ERW are due to its rich H_2_ content, enabling it (1) to reduce cytotoxic oxygen radicals in cultured cells and protect ischemia-reperfusion injury in rats [14]; (2) to suppress the expression of proinflammatory cytokines by regulating gene expression [15]; (3) to stabilize atherosclerotic plaques [16]; (4) to protect from peroxynitrite derived from nitric oxide [17]; and (5) to reduce the number of injuries caused by hypoxia/reoxygenation [18] and cecal ligation [19]. Regarding the anticancer effects of ERW, it was reported that it reduced tumor angiogenesis by downregulation of VEGF gene transcription and protein secretion through inactivation of ERK [20]; it enhanced apoptosis in human leukemic HL-60 cells [21]; it inhibited colony formation and cell proliferation of human tongue squamous cell carcinoma-derived cell line HSC-4 but not of normal human tongue epithelial-like cells DOK [22]; it inhibited proliferation and invasion of the human fibrosarcoma HT-1080 cells [22]; it strongly prevented (when supplemented with synthesized Pt nanoparticles) the transformation of BALB/c 3T3 cells by 3-methyl cholanthrene as an initiation substance, followed by treatment with phorbol-12-myristate-13-acetate as a promotion substance [23]. While the* in vitro* results appeared to support the inhibition of cell proliferation by ERW, the analysis of its* in vivo* effects on tumor growth could better sustain its potential medical applications. The transgenic BALB-*neu*T mouse represents a valuable tumor model for HER2 breast carcinoma, closely reproducing some features of mammary carcinogenesis in women [24]. Here we evaluated the* in vitro* and* in vivo* effects of ERW on cancer cell growth, together with the analysis of involved molecular mechanisms.

## 2. Materials and Methods

### 2.1. Equipment

An alkaline ionized, seven electrode plates platinum-plated, water generator was used to produce ERW (Chanson “Miracle Max” Water Ionizer, Taiwan). The generator is provided with high efficiency activated carbon and works with tap water. The ERW produced by the generator has low ORP (-772 ± 51 mV), elevated concentration of dissolved hydrogen (H_2_, 1327 ± 158), and an alkaline pH (10.8).

### 2.2. Reagents

Sulforhodamine B (SRB) was purchased from Sigma-Aldrich (Saint Louis, MO, USA). Antibodies against AKT and phospho-AKT (Ser473) were obtained from Cell Signaling Technology (MA, USA). Antibodies against Bax and Bcl-2 were from BD Pharmigen (BD Biosciences, San Josè, CA, USA). Antibodies against ERK1/2 (C-14), phospho-ERK (E-4), PARP-1, and p53 (DO-1) were obtained from Santa Cruz Biotechnology (Santa Cruz, CA, USA). Anti-ErbB2 antiserum was provided by Dr. M.H. Kraus (University of Alabama, Birmingham, USA). A rabbit polyclonal antibody against actin and goat anti-mouse or anti-rabbit IgG peroxidase-conjugated secondary antibodies were from Sigma-Aldrich.

### 2.3. Cell Lines and Treatments

BALB-*neu*T mammary cancer cells (H-2^d^) (TUBO) that overexpress activated rat ErbB2/*neu* were kindly provided by Professor G. Forni and Professor F. Cavallo (University of Torino, Italy) [25] and were maintained in DMEM (Dulbecco's modified Eagle's medium) liquid (Aurogene, Rome, Italy) containing 20% fetal bovine serum, 100 U/ml penicillin and 100 *μ*g/ml streptomycin (complete medium). Human (MCF-7 and MDA-MB-453) breast cancer cell lines were maintained in DMEM liquid (Aurogene, Rome, Italy) containing 10% fetal bovine serum, 100 U/ml penicillin, and 100 *μ*g/ml streptomycin (complete medium). Cells were grown at 37°C in a humidified incubator with an atmosphere of 5% CO_2_. During the experiments, cells were cultured with DMEM, high glucose (Gibco, Thermo Fisher Scientific, Waltham, MA, USA) prepared by dilution of 10x DMEM with ERW, designated as ERW treatment (ERW: ORP -450 ± 37 mV, pH 7.3 ± 0.1, H_2_ = 751 ± 136), or cultured with DMEM prepared by dilution of 10x DMEM with autoclaved ERW, designated as autoclaved treatment (autoclaved ERW: ORP +50 ± 6 mV, pH 7.3 ± 0.1, H_2_ = 0). We used DMEM liquid (Aurogene, Rome, Italy) only as a positive control medium to monitor the cell growth, because breast cancer cells were routinely maintained in this medium (CTR: ORP +50 ± 8 mV, pH 7.3 ± 0.1, H_2_ = 0). All culture media were enriched with 10% FBS (MCF-7, MDA-MB-453) or 20% FBS (TUBO). All electrolyzed alkaline water (ERW and autoclaved ERW) was neutralized at pH 7.0-7.4 by adding 3 ml of DMEM 10x, 0.5 ml of 1 M HEPES buffer (pH 5.3, Sigma-Aldrich) and 0.3 ml of penicillin-streptomycin every 27 ml of ERW/autoclaved ERW, before use. Treatments were replenished every 24 hours. We compared through all the experiments the effects of ERW with the effect of autoclaved ERW. Thus, the autoclaved ERW with positive ORP and no concentration of dissolved hydrogen was our control for ERW treatment, since the differences between the effect of ERW and autoclaved ERW can be attributed to the unique characteristics possessed by ERW. Indeed, the ERW treatment possesses a negative ORP and an elevated high concentration of dissolved hydrogen, while the autoclaved ERW treatment possesses a positive ORP and no concentration of dissolved hydrogen.

### 2.4. Cell Proliferation Analysis by Sulforhodamine B (SRB) Assay

Cells were seeded at 5 × 10^3^/well in 96-well plates. After 24 hours, cells were treated with ERW, autoclaved ERW and control medium (CTR), and incubated for 24-72 hours. The assay was then performed as previously described [26]. The percentage survival of the cultures treated with ERW or autoclaved ERW was calculated by normalizing their OD values to those of control cultures (CTR) [27]. The experiments were performed in triplicate and repeated six times.

### 2.5. Cell Viability Analysis by Trypan Blue Exclusion Test

Breast cancer cells (5 × 10^4^ cells/well) were seeded in 24-well plates. After 24 hours, cells were treated with ERW, autoclaved ERW and control medium (CTR), and incubated for 24-72 hours. Media were replaced every 24 hours and cell density was determined. After treatment, adherent and suspended cells were harvested and stained with trypan blue (Sigma-Aldrich) and counted. The experiments were performed in triplicate and repeated six times. The percentage of cell death was determined compared to the total number of cells as previously described [28].

### 2.6. ERW Treatment of BALB-neuT Mice

Transgenic BALB-*neu*T male mice were mated with BALB/c females (H-2^d^; Charles River, Calco, Italy) in the animal facilities of Tor Vergata University. Founder male BALB-*neu*T mice were kindly provided by Professor G. Forni and Professor F. Cavallo (University of Turin, Italy) [25]. Progenies (48 mice) were confirmed for the presence of the transgene by PCR [25]. Sixteen individually tagged virgin females were used in this study. Mice were bred under pathogen-free conditions and handled in compliance with European Union and institutional standards for animal research.

Groups of female BALB-*neu*T mice (8 mice per group) were treated with ERW water or natural water since their weaning and up to 33 weeks of age. The ERW water was changed every three hours during the day while the night was left to mice available* ad libitum*. Mice were sacrificed at the first signs of distress. Investigation has been conducted in accordance with the ethical standards and according to the Declaration of Helsinki. A veterinary surgeon was present during the experiments. The animal care both before and after the experiments was performed only by trained personnel. The work was conducted with the formal approval of the local animal care committees (institutional and national), and animal experiments have been registered as legislation requires (Authorization from Ministry of Health n° 875/2016-PR, 16/09/16).

### 2.7. Analysis of ERW Antitumor Activity In Vivo

Mammary glands were checked weekly and tumors recorded at 2-3 mm in diameter. Tumor growth was monitored until all mammary glands displayed a palpable tumor or tumor mass exceeding 20 mm in diameter. At this point mice were sacrificed. The time of tumor appearance was recorded and tumor multiplicity for each treatment group was expressed as mean ± standard deviation (SD) [29].

### 2.8. Evaluation of Hematological and Clinical Chemistry Parameters

Blood samples were collected in animals under i.p. anesthesia (20 *μ*l/g.b.w. of 1.2% Avertin-2,2,2- tribromoethanol, 2.4% 2-methyl-2-butanol; Sigma-Aldrich). Hematological parameters were analyzed using the commercially available automated cell counter “Drew3” (BPC BioSed, Italy), after collecting 20 *μ*l of whole blood in K2EDTA microvacutainers (Boston, Dickinson and Company, USA). Peripheral blood smears were prepared employing the differential staining Diff-Quick (Dade SpA, Italy) and analyzed for cytomorphological parameters under optical microscopy. The complete blood count (CBC) includes hemoglobin (HGB), hematocrit (HCT), red blood cell (RBC) count, white blood cell (WBC) count, platelet count (PLT), red cell distribution width (RDW), mean corpuscular volume (MCV), mean platelet volume (MPV), mean corpuscular hemoglobin (MCH), mean corpuscular hemoglobin concentration (MCHC), lymphocytes (LYMF), midrange cells (MID), and granulocytes (GRAN). Blood samples were collected in SST microtainers (Serum Separator Tube; Boston, Dickinson and Company) for serum protein electrophoresis and centrifuged at 13.000 rpm for 7 min. For the measurement of blood glucose, mice were placed in fasting three hours before blood sampling. Glucose (GLU) was measured using the automatic analyzer Keylab (BPC BioSed s.r.l., Rome, Italy).

### 2.9. Western Blotting Analysis

1 × 10^6^ cells were seeded in 100-mm tissue culture dishes 24 hours prior to the addition of ERW and autoclaved ERW treatments. Media were replaced every 24 hours. Western blotting analysis was performed as previously described [26]. 50 *μ*g of cell lysates was resolved in 10% SDS-PAGE and then transferred to nitrocellulose membranes. After blocking, the membranes were incubated with specific primary antibodies at 1-2 *μ*g/ml concentrations overnight at 4°C. After being washed, the filters were incubated with goat anti-mouse or anti-rabbit IgG, peroxidase-conjugated antibodies and developed by chemiluminescence as previously described [30]. A densitometric analysis of autoradiographic bands was performed with Image J software 1.42q (National Institutes of Health, Bethesda, MD, USA) after blot scanning and expressed as bar graphs in the figures.

### 2.10. Fluorescence-Activated Cell Sorting (FACS) Analysis

Asynchronized, log-phase growing cells (60% confluent, approximately 2.5 × 105/well in 6-well plates) were treated with ERW, autoclaved ERW or control medium (CTR). Media were replaced every 24 hours. Fluorescence-activated cell sorting (FACS) analysis was performed as previously described using a FACSCalibur cytometer with CellQuest Pro 5.2 software (BD Biosciences, San Jose, CA) [26, 31]. A minimum of 20000 events was collected. The experiments were repeated three times

### 2.11. Statistical Analysis

Data distribution of cell survival, cell death, or FACS analyses was preliminarily verified by the Kolmogorov-Smirnov test, and datasets were analyzed by one-way analysis of variance (ANOVA) followed by Newman-Keuls test as previously described [26, 31]. Differences in the intensity of immunoreactive bands between ERW and autoclaved ERW were evaluated by a two-tailed Student's t-test. Values with p<0.05 were considered significant. For the hematological and clinical chemistry parameters, only the parameters outside the reference values were analyzed by a two-tailed Student's t-test comparing those of control mice with the ERW-treated mice. Survival curves and tumor multiplicity were estimated using the Kaplan-Meier method and compared by the log-rank test (Mantel-Cox) with calculation of the SD according to the method of Greenwood. Differences were regarded as significant when p value was <0.05 as previously described [29].

## 3. Results

### 3.1. ERW Inhibits Human and Mouse Breast Cancer Cells Survival

We first evaluated the* in vitro* effect of ERW on the growth of human breast cancer cell lines (MCF-7 and MDA-MB-453), characterized by different expression of estrogen (ER), progesterone (PR), and ErbB2 receptors, and of tumor suppressor p53 [32–36] (Table 1). In addition, we employed the mouse breast cancer cell line TUBO, derived from BALB-*neu*T mice that overexpresses activated rat ErbB2/*neu*.

**Figure 1:**
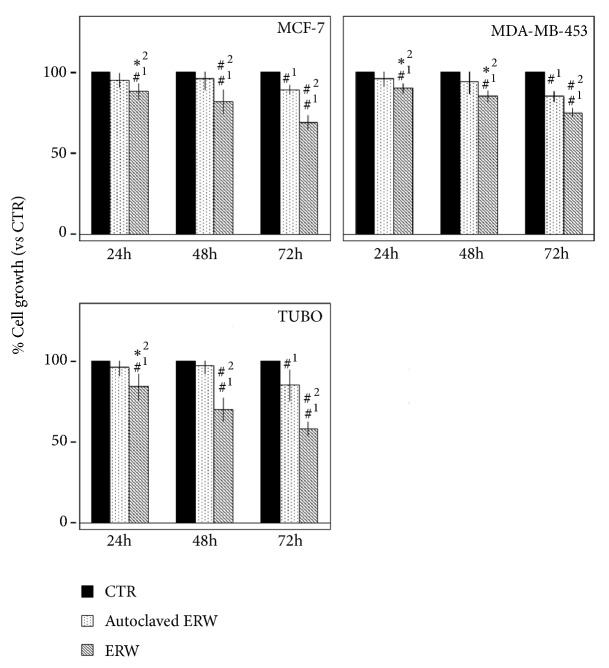
**Effect of ERW on breast cancer cell lines survival**. The survival of human (MCF-7, MDA-MB-453) and mouse (TUBO) breast cancer cell lines was assessed by the SRB assay after 24, 48, and 72 hours of treatment with ERW, autoclaved ERW, and control medium (CTR). The percentage of cell growth treated with ERW and autoclaved ERW was calculated by normalizing the OD value to that of the control cultures (CTR). The results are expressed as the mean ± SD of six independent experiments performed in triplicate (*∗*p ≤ 0.01, ^#^p ≤ 0.001 compared with the cultures treated with ^1^CTR and ^2^autoclaved ERW).

Cell growth of human (MCF-7, MDA-MB-453) and mouse (TUBO) breast cancer cells was quantified after treatment with ERW, autoclaved ERW, and CTR (used as positive control cultures to monitor the cell growth) for 24, 48, and 72 hours by the SRB assay. The effects of autoclaved ERW achieved statistical significance only at 72 hours compared to CTR treatment in all cell lines (p<0.001). Conversely, the effect of ERW reached statistical significance at all times analyzed as compared to CTR treatment in all cell lines (p<0.001). In addition, ERW treatment had greater cell survival inhibition than autoclaved ERW at all times in all cell lines analyzed (p<0.01) (Figure 1).

**Figure 2:**
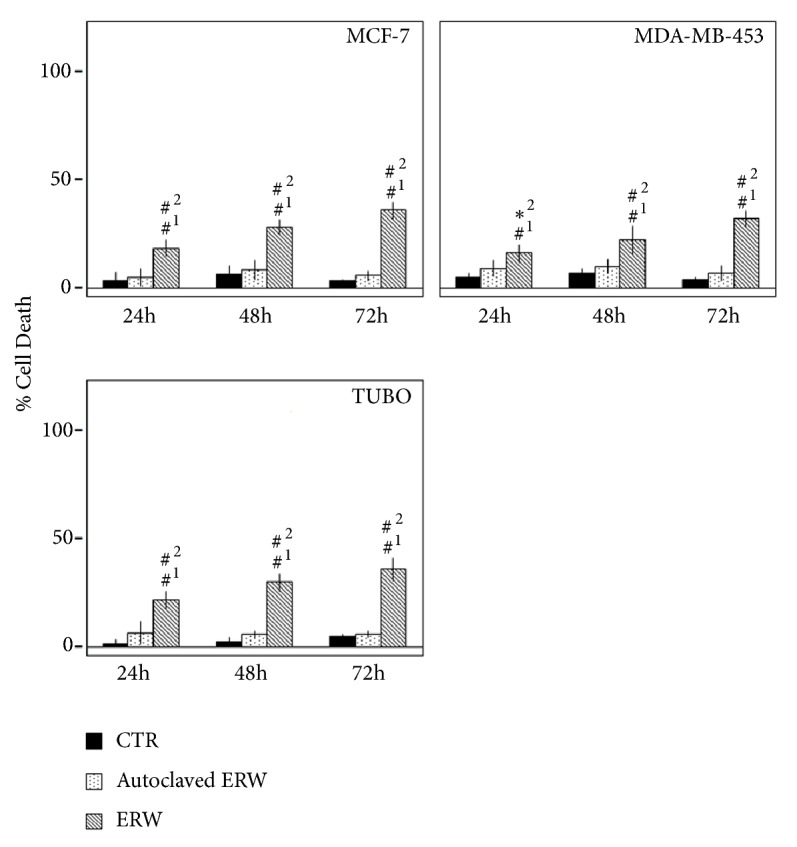
**Effect of ERW on death of breast cancer cell lines**. Trypan blue exclusion test was performed to determine the percentage of cell death of breast cancer cells treated with ERW, autoclaved ERW, or control medium (CTR) after 24, 48, and 72 hours of treatment. The results are expressed as the mean ± SD of six independent experiments performed in triplicate (*∗*p ≤ 0.01, ^#^p ≤ 0.001 compared with the cultures treated with ^1^CTR and ^2^autoclaved ERW).

The dye exclusion test was used to determine the number of death breast cancer cells after ERW, autoclaved ERW and CTR treatments. The cytotoxic effect of ERW was time-dependent. Autoclaved ERW had no significant effect as compared to CTR. Conversely, the percentage of cell death upon ERW treatment was 22, 34, and 43 for MCF-7 (p<0.001); 16, 22, and 32 for MDA-MB-453 (p<0.001); 26, 36, and 43 for TUBO (p<0.001) cells (Figure 2). In addition, the effects obtained with ERW treatment gained statistical significance as compared to autoclaved ERW treatment at all times analyzed (MCF-7 and TUBO: p<0.001; MDA-MB-453: p<0.01 at 24 h and p<0.001 at 48 and 72 h) (Figure 2).

### 3.2. ERW Delays Mammary Tumor Growth in BALB-neuT Mice

The* in vivo* antitumor activity of ERW was analyzed in BALB-*neu*T mice transgenic for the* neu* oncogene. This mouse model represents an aggressive ErbB2/*neu*-mediated mammary carcinogenesis. Indeed, BALB-*neu*T mice develop atypical hyperplasia of the mammary gland at 6 weeks of age and* in situ* and invasive carcinoma at 11 and 16 weeks of age, respectively [25]. Groups of female BALB-*neu*T mice (8 mice per group) were treated with ERW or natural water (control) since their weaning and up to 33 weeks of age.

**Figure 3:**
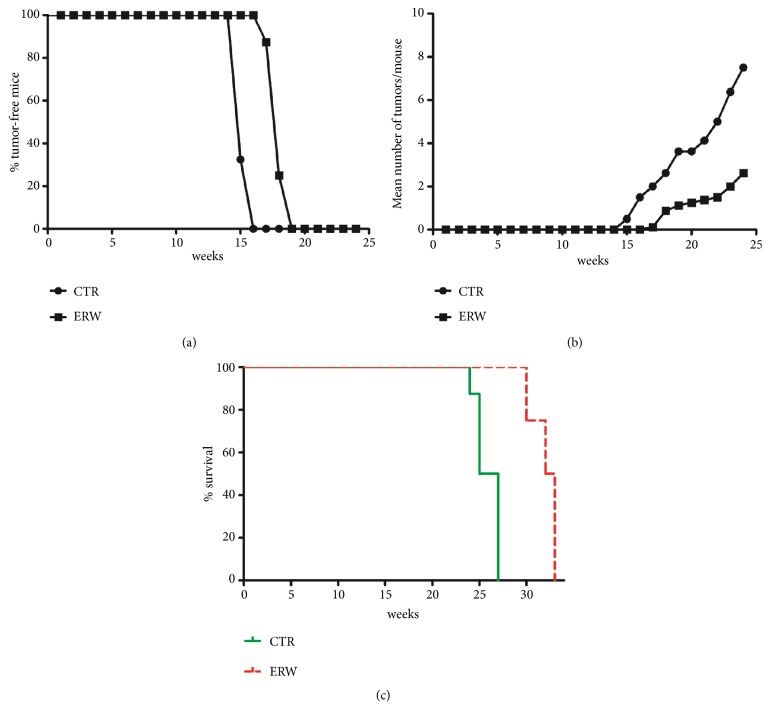
**Delay of tumor growth in BALB-*neu*T mice treated with ERW. (a)** Differences in the time of tumor appearance.** (b)** Differences in tumor multiplicity expressed as mean of incidental tumors.** (c)** Differences in the mean survival duration of BALB-*neu*T mice treated with ERW or natural water (CTR).

All BALB-*neu*T mice treated with natural water had palpable tumors by week 16, with a tumor multiplicity of 1.5. By contrast, none of the ERW-treated mice exhibited signs of tumor growth at week 16, indicating specific interference of ERW water with tumor development (p<0.001) (Figures 3(a) and 3(b)). Indeed, in the ERW-treated group palpable tumors appeared and affected all mice only at week 19. In addition, at week 24, when the first control mouse was sacrificed for the excessive size of its tumors, the multiplicity of tumors in control mice was 7.5, while that of ERW-treated mice was 2.63 (p<0.001) (Figures 3(a) and 3(b)). Three and four control mice were euthanized at 25 and 27 weeks, respectively. At 27 weeks of age, when all control mice were sacrificed, ERW mice were still all alive. ERW mice were euthanized for the excessive size of at least one tumor per mice at 30 weeks (2 mice), at 32 weeks (2 mice), and at 33 weeks (4 mice), when the experiment was completed.

Our results indicate that ERW induced a significant prolongation of tumor-free survival and a reduction of tumor multiplicity (Figures 3(a) and 3(b)). Furthermore, our results indicated a specific interference of ERW with tumor growth in BALB-*neu*T mice (Figure 3(c)). The increase in the median survival of ERW-treated mice was significant compared to control mice (32.5* versus* 26 weeks, p=0.0001) (Figure 3(c)). The relative risk of developing tumors in the control mice was 22.25 as compared to ERW-treated mice (Table 2).

### 3.3. Hematological and Clinical Chemistry Parameters in Mice Administered with ERW

To determine whether ERW induced changes in hematological and clinical chemistry parameters, we measured complete blood count, cholesterol, triglycerides, serum glutamic oxaloacetic transaminase, serum pyruvic transaminase, glucose, and blood urea nitrogen, in BALB-*neu*T mice (8 per group) at 7/11/17/23 weeks of age and at the end of the protocol. Values of individual mice were very heterogeneous within each group. No significant differences were observed between the parameters measured in each group of mice at 7 weeks of age. On the other hand, at 11 weeks of age ERW-treated mice showed a decrease in the total number of white blood cells (reference values 5-12 × 10^3^/mm^3^; 3.9* versus* 7.5, p<0.001), lymphocytes (reference values 4-10 × 10^3^/mm^3^; 2.6* versus* 4.6, p<0.001), and triglycerides (reference values 80-200 mg/dl; 54.0* versus* 122.6, p<0.001) compared to control mice. At this stage, the control mice displayed a slight decreased level of mean corpuscular hemoglobin (reference values 15-19 pg; 13.9* versus *15.4, p<0.001) and mean corpuscular hemoglobin concentration (reference values 34-39 g/dl; 30.4* versus* 34.4, p<0.001) compared to ERW-treated mice. Only a significant decrease of triglycerides in control mice compared to ERW-treated mice (52.8* versus* 86.7, p<0.05) was observed after 17 weeks of age. In addition, at 23 weeks of age ERW-treated mice displayed a decreased level of mean corpuscular volume (reference values 43-58 *μ*m^3^; 41.9* versus* 44.1, p<0.01) as compared to control mice. It is important to note that control mice showed a marked decrease in the percentage of lymphocytes (reference values 50-75%; 34.2* versus* 62.2, p<0.05) and a marked increase in the percentage of granulocytes (reference values 15-40%; 59.1* versus *30.3, p<0.05) as compared to ERW-treated mice, at the end of treatment. Conversely, the percentages of lymphocytes and granulocytes in the ERW-treated mice fell within the reference values (Table 3). Collectively, these results demonstrated that ERW did not affect hematological and clinical chemistry parameters and that the alterations observed in the control and ERW-treated mice are likely associated with tumor growth.

### 3.4. ERW Decreases ErbB2/neu Expression and Impairs Phospho-ERK1/ERK2 and AKT Phosphorylation

**Figure 4:**
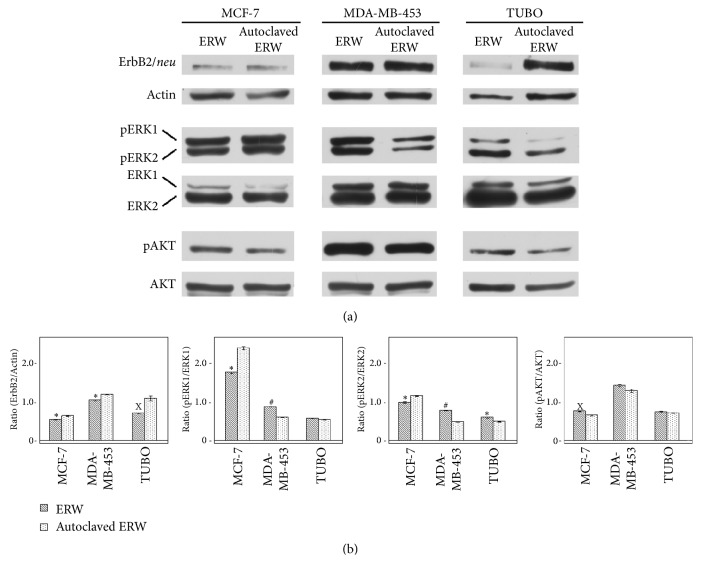
**Effect of ERW on the expression and activation of ErbB receptor and prosurvival signaling pathway molecules. **Western blotting analysis was performed on breast cancer cell lines that were treated with ERW and autoclaved ERW for 48 hours.** (a)** The levels of pERK1 and pERK2 proteins as well as pAKT proteins were compared with the total ERK and AKT protein levels, respectively. Actin was used as an internal control.** (b)** Densitometric ratios and statistical analysis are reported. Data are expressed as the mean ± SD of two independent experiments. The statistical significance of the effects obtained with ERW was calculated* versus* those obtained with autoclaved ERW (^x^p ≤ 0.05, *∗*p ≤ 0.01, and ^#^p ≤ 0.001).

ErbB2/*neu* plays a critical role in breast cancer development [37]. Therefore, the interference of ERW treatment to the expression of ErbB2/*neu* and activation of ErbB2/*neu* signaling (pERK1/ERK2 and AKT) has been investigated. ERW decreased ErbB2/*neu* expression in MCF-7 (p=0.008), MDA-MB-453 (p= 0.002), and TUBO cells (p=0.014) as compared to autoclaved ERW (Figures 4 and S1). ERW treatment impaired ERK1 and ERK2 phosphorylation as compared to autoclaved ERW in MCF-7 cells (p=0.002 and p=0.0048, respectively). Conversely, ERW increased ERK1 and ERK2 phosphorylation in MDA-MB-453 (p=0.0001 and p=0.0004) cells and ERK2 in TUBO cells (p=0.006) (Figures 4 and S1). In addition, ERW treatment increased AKT phosphorylation at Ser473 as compared to autoclaved ERW only in MCF-7 (p=0.018) (Figures 4 and S1).

### 3.5. ERW Induces Apoptosis of Breast Cancer Cell Lines

**Figure 5:**
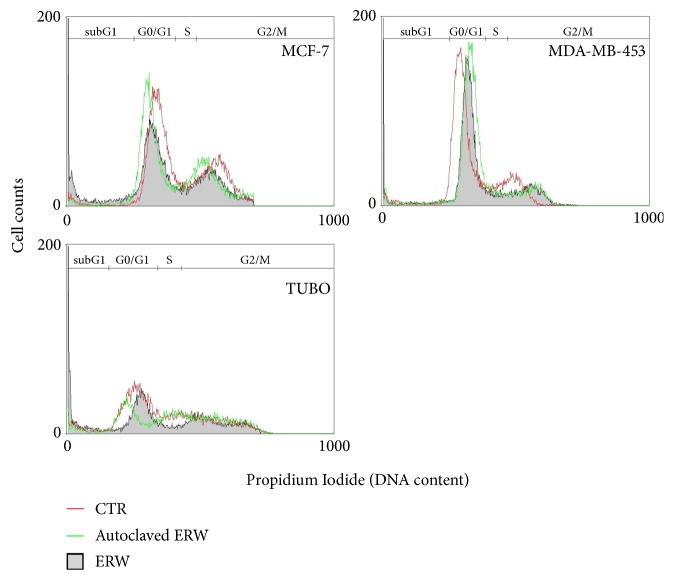
**Effect of ERW on cell cycle distribution**. FACS analysis of DNA content was performed on asynchronized log-phase growing breast cancer cells treated for 72 hours with ERW, autoclaved ERW, or control medium (CTR). A representative experiment is shown in the figure.

**Figure 6:**
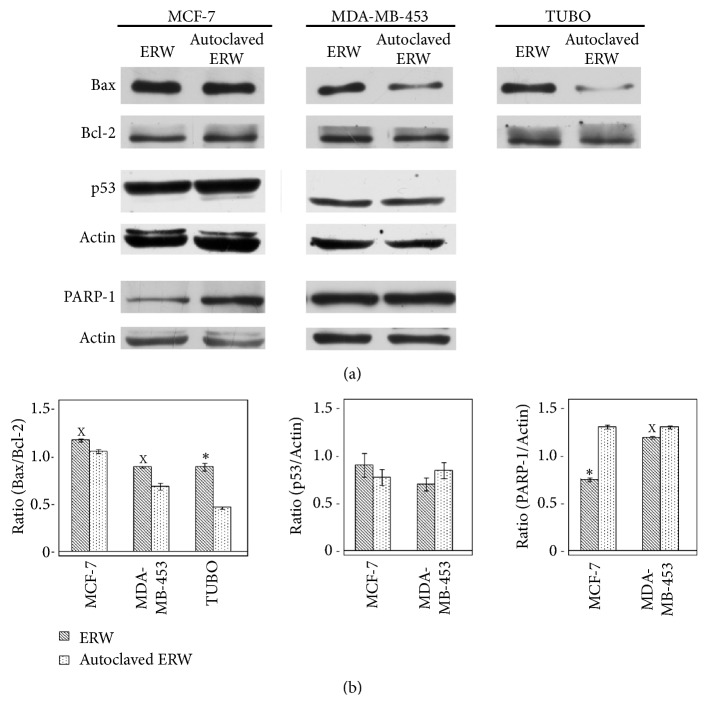
**Effect of ERW on apoptosis in breast cancer cell lines. (a)** The expression of Bax, Bcl-2, p53, and PARP-1 was assessed by Western blotting analysis in breast cancer cell lines that were treated with ERW and autoclaved ERW for 48 hours. Actin was used as an internal control.** (b)** The densitometric ratios between Bax and Bcl-2, p53 and actin, PARP-1 and actin, and statistical analysis are reported. Data are expressed as the mean ± SD of two independent experiments. The statistical significance of the effects obtained with ERW was calculated* versus* those obtained with autoclaved ERW (^x^p ≤ 0.05, *∗*p ≤ 0.01).

To determine ERW-mediated effects on cells cycle and apoptosis of breast cancer cells, FACS analysis of DNA content was performed.  Figure 5 shows a representative experiment in which the effect of ERW on DNA content was compared to that obtained with autoclaved ERW and with control cultures. The mean results of three independent experiments are reported in Table 4. Our results demonstrated that ERW treatment induced an increase in the percentage of cells in the sub-G1 phase as compared to autoclaved ERW in MCF-7 (p<0.001), MDA-MB-453 (p<0.001), and TUBO (p<0.01) cells. The increase in the percentage of cells in sub-G1 phase was paralleled by the decrease of the percentage of cells in G0/G1 phase in MCF-7 (p<0.01), MDA-MB-453 (p<0.05), and in S and G2/M phases in TUBO cells (p<0.05) (Table 4 and Figure 5). To corroborate that the ERW increase in the percentage of cells in sub-G1 phase was partially caused by the induction of apoptosis, the Bax/Bcl-2 expression ratio was analyzed by Western blotting. ERW treatment increased the Bax/Bcl-2 ratio in both human and mouse cell lines compared to autoclaved ERW treatment (MCF-7, p=0.02; MDA-MB-453, p=0.01; TUBO, p=0.0046) (Figures 6 and S1). In addition, ERW and autoclaved ERW did not modulate the expression of p53 in MCF-7 cell line. Decreased PARP-1 expression upon ERW treatment was detected both in MCF-7 (p=0.001) and MDA-MB-453 (p=0.01) cells as compared to autoclaved ERW treatment (Figures 6 and S1).

## 4. Discussion

ERW has shown to possess (1) suppressive effects against oxidative stress, granting DNA protection [4, 9]; (2) carbon tetrachloride-induced liver damage reduction [38]; (3) alloxan-induced type 1 diabetes modulation [4, 39]; (4) hemodialysis-induced oxidative stress reduction during end-stage renal disease [40, 41]; (5) nephroprotective effects on cisplatin-induced kidney damage in mice [42]; and (6) suppressive effects on lipopolysaccharide-induced neuroinflammation [43]. Furthermore, Pt nanoparticles produced by electrode plates during electrolysis and visualized by transmission electron microscopy [1] can play an additional role in the ROS-scavenging activity of ERW [8].

Previous studies have reported anticancer effects of ERW on human lung adenocarcinoma cell line A549 [20], human leukemic HL-60 cells [21], squamous cell carcinoma-derived cell line HSC-4 [22], fibrosarcoma HT-1080 cells [8, 22, 44], human leukemic monocyte lymphoma cell line U937 [45], and BALB/c 3T3 cells [23]. Despite the possibility that ROS might play an underlying role in the oxidative damage process that is closely related to many chronic and inflammatory diseases including cancer, no effects on tumor growth have been previously studied in* in vivo* models using ERW water.

The present study has added information of ERW anticancer activity in never before tested human and mouse breast cancer cell lines, including BALB-*neu*T mammary cancer cells (H-2^d^) (TUBO), MCF-7, and MDA-MB-453. We demonstrated that ERW treatment inhibited cell survival, induced apoptosis of breast cancer cells, decreased ErbB2/*neu* expression, and impaired pERK1/ERK2 activation, as previously reported in another cell line [20]. The effect on ErbB2/*neu* expression could be linked to the antioxidant properties of ERW. It has been described that ErbB2 and ErbB3 expression can be induced by ROS and that ErbB2-dependent signaling contributes to antioxidant defenses [46, 47]. In addition, it has been described that flavonoids act as antioxidants and decreases the expression of ErbB2 and ErbB3 proteins in HT-29 human colon cancer [48]. Furthermore, Pt nanoparticles present in ERW can play a synergic effect in the viability reduction of tumor cells [49]. It has been suggested that Pt nanoparticles may serve as a reservoir for Pt ions that can induce DNA damage in cancer cells [50].

In addition, ERK1/2 MAP kinase signaling pathway is abnormally and frequently activated in human cancer [51] and plays a key role in cell proliferation. Here, we demonstrated that ERW down-regulates MAP kinases activity as compared to the autoclaved ERW in MCF-7 cells. The effect of ERW on ERK1/2 was opposite in the other cell lines. This opposite effect might be due to the expression of alternative pathways activation in different cell lines and might mirror the tumor heterogeneity occurring in human tumors. On the other hand, these cell lines were sensitive to the proapoptotic effect of ERW as well, thus suggesting that MAPK activation could trigger apoptosis in these cell lines. Indeed, it was reported that MAPK/ERK activation could protect or contribute to cell death depending on the specific context [52, 53].

In addition, we showed that ERW treatment induced a slight phosphorylation at Ser473 in MCF-7 cells. It has been reported that phosphorylation at Thr308 is necessary and sufficient for AKT activation, but the second phosphorylation at Ser473 is required for maximal activation of AKT [54]. AKT activation plays a key role in cell survival and tumor growth. However, despite AKT phosphorylation, we found that ERW inhibited cells survival, increased cell death, and inhibited ERK activation in this cell line. The contribution of the activation of AKT to cell death is not fully clear. However, a crosstalk between PI3K-AKT and Ras-Raf-MEK-ERK pathways has been reported. AKT inactivated c-Raf and B-Raf by directly phosphorylating them and thus inhibiting the MEK-ERK pathway in different cell lines [55–58]. In addition, different apoptotic stimuli, such as curcumin, taxol, etoposide, and staurosporine, are able to induce apoptosis and at the same time AKT phosphorylation in Ser473 [59]. Furthermore, it has been shown that AKT can induce phosphorylation of histone H2A and CDK2, signals that could favor the activation or maintenance of apoptosis [60, 61].

We also demonstrated that ERW induces apoptosis both in human and mouse breast cancer cell lines. In particular, we showed that the ERW-mediated increase in the percentage of cells in sub-G1 phase was caused by the induction of apoptosis, as confirmed by the increase in the ratio of the proapoptotic protein Bax to the antiapoptotic protein Bcl-2. It was previously reported that ERW induced apoptosis through the upregulation of Bax and downregulation of Bcl-2 in human leukemic HL-60 cells [21]. Of note, ERW induced apoptosis in both human and mouse breast cancer cell lines and ERW induced apoptosis was not dependent on p53 expression since ERW treatment triggered apoptosis also in MDA-MB-453 carrying mutated p53. In addition, cell death induced by ERW treatment was not dependent on ER and PR status. Our results showed that although the Bax/Blc-2 ratio increased in ERW-treated cultures as compared to autoclaved ERW, we did not observed the cleavage of PARP-1. However, we observed a significant downregulation of the expression of total PARP-1 in MCF-7 and MDA-MB-453 cells. PARP-1 is overexpressed in different types of cancer, including prostate, pancreatic, and breast, and it has been reported that decreased expression of PARP-1 inhibited cell proliferation and induced apoptosis in pancreatic cancer cells [62]. In addition, the degradation of PARP-1 mediated by caspase-independent ubiquitylation which has a role in apoptosis, necrosis, and other cellular processes has been reported [63–66]. Accordingly, the observed decrease of PARP-1 expression might confirm apoptosis activation.

This is the first time that an* in vivo* mouse model (BALB-*neu*T) is used to show the direct effects of ERW on cancer. Previous* in vivo* studies with ERW focused on other factors: nephroprotective effect of ERW against cisplatin-induced kidney toxicity and oxidative damage in mice [42] and antioxidant and anti-inflammatory effects of hydrogen-rich water in alleviating ethanol-induced fatty liver in mice [67]. Our* in vivo* study showed that BALB-*neu*T mice that drank ERW delayed the development of tumors compared to the control. In addition, ERW induced a significant prolongation of tumor-free survival and a reduction in tumor multiplicity. The increase in the median survival of ERW-treated mice was highly significant as compared to control mice (p=0.0001) and the relative risk of developing tumors in the control mice was 22.25 as compared to the ERW-treated mice. The observed inhibition of* in vivo* growth could be due to the direct effect of ERW on ErbB2 receptor that is abnormally activated in this mouse model, the impairment of its downstream effector MAPK ERK, and the induction of apoptosis. The overexpression of ErbB2 was reported to inhibit both the intrinsic and extrinsic pathway of apoptosis in breast cancer [68].

Furthermore, biochemical parameters did not differ significantly between ERW-treated group and control group. However, a slight difference was found on white cells count between day 11 and 17 (most lymphocytes and neutrophils). This modification is likely associated with advanced neoplastic stage in control mice. In fact ERW delayed the appearance of the tumor in the ERW-treated BALB-*neu*T mice as compared to control mice. A feasible mechanism could be also due to a decrease in the inflammatory state that would guarantee ERW-treated BALB-*neu*T mice a better immune surveillance towards the onset of the tumor.

Overall, our* in vitro* and* in vivo* results suggest a role of ERW in inhibiting cancer cell growth. Further studies are warranted to investigate ERW potential use in breast cancer treatment.

## 5. Conclusions

In this study, we show that ERW treatment inhibited cell survival, induced cell apoptosis, decreased ErbB2/*neu* expression, and impaired pERK1/ERK2 activation in breast cancer cells. In addition, our* in vivo* results showed that ERW induced a significant prolongation of tumor-free survival and a reduction in tumor multiplicity in transgenic BALB-*neu*T mice. Overall, these results suggest a potential beneficial role of ERW in inhibiting cancer cells growth. Further studies are warranted to investigate ERW potential use in breast cancer treatment.

## Figures and Tables

**Table 1:** Molecular features of employed human breast cancer cell lines.

**Cell line**	**ER** ^†^	**PR**	**ErbB2**	**p53**	**References**
**MCF-7**	+	+	+	+^WT^	[32–35]
**MDA-MB-453**	-	-	+/++	+^M^	[32, 33, 35, 36]

^†^Estrogen and progesterone receptors (ER/PR) status, ErbB2 expression, p53 protein levels, and mutational status (M: mutated protein; WT: wild-type protein) are indicated.

**Table 2:** Analysis of the survival of BALB-*neu*Tmice after treatment with ERW by the log-rank test (Mantel-Cox).

**Variable**	**Contrast**	**Hazard Ratio**	**95**%** Hazard Ratio Confidence Limits**		**Median Survival (Weeks)**	**p Value**
			Lower	Upper		
Treatment	CTR *vs. *ERW	22.25	4.577	108.2	26 *vs.* 32.5	0.0001

CTR: control mice; ERW: mice treated with ERW.

**Table 3:** Complete blood count (CBC) and clinical chemistry parameters in BALB-*neu*T mice treated with ERW or natural water (CTR).

**CBC**	**Reference values**	**7 weeks of age**		**11 weeks of age**		**17 weeks of age**		**23 weeks of age**		**End of treatment**	
		**CTR**	**ERW**	**CTR**	**ERW**	**CTR**	**ERW**	**CTR**	**ERW**	**CTR**	**ERW**
WBC (10^3^/mm^3^)	5.0-12.0	5.1	5.5	7.5	3.9^**#**^	5.2	5.2	5.3	3.8	5.2	10.5
LYM (10^3^/mm^3^)	4.0-10.0	3.2	3.8	4.6	2.6^**#**^	2.9	3.4	3.0	2.1	1.4**∗**	6.5
GRAN (10^3^/mm^3^)	0.5-6.0	1.5	1.3	2.2	1.0	1.8	1.4	1.7	1.2	2.9	2.5
MID (10^3^/mm^3^)	0-2.0	0.5	0.4	0.6	0.4	0.5	0.5	0.6	0.5	0.4	1.4
LYM (%)	50.0-75.0	63.1	68.4	62.7	66.2	55.5	64.9	57.0	54.3	34.2^**x**^	62.2
GRAN (%)	15.0-40.0	28.9	23.6	30.0	26.3	35.4	27.5	33.1	35.0	59.1^**x**^	30.3
MID (%)	5.0-15.0	8.0	8.0	7.3	7.5	9.1	7.6	9.9	10.8	6.8	7.6
HCT (%)	35.0-60.0	53.5	52.2	57.8	50.8	54.3	54.6	55.6	47.5	50.2	42.7
MCV (*μ*m^3^)	43.0-58.0	46.2	46.0	45.7	44.8	45.6	43.1	44.1	41.9**∗**	42.8	39.9
RBC (10^6^/mm^3^)	6.0-12.0	11.1	11.3	12.0	11.3	11.9	12.0	11.8	11.3	11.7	10.4
HGB (g/dl)	9.0-19.0	16.9	15.4	17.5	17.5	17.3	18.9	18.7	16.6	17.8	15.1
MCH (pg)	15.0-19.0	13.9	13.6	13.9^**#**^	15.4	14.6	15.0	15.3	14.7	15.2	14.5
MCHC (g/dl)	34.0-39.0	29.8	29.6	30.4^**#**^	34.4	32.0	35.2	34.7	35.0	35.5	36.3
RDW (%)	0-99.9	16.4	16.5	15.3	14.5	14.9	15.9	15.9	15.5	15.6	17.9
MPV (*μ*m^3^)	4.0-7.0	5.8	5.8	5.8	6.0	5.7	5.9	5.8	5.8	6.4	6.2
PLT (10^3^/mm^3^)	250-1200	665.0	693.8	796.4	630.3	713.8	923.8	823.0	497.8	860.5	859.0

**Clinical Chemistry**											

CHOL (mg/dl)	50-120	78.5	77.0	75.4	57.0	64.4	68.0	59.2	60.0	68.0	91.7
TRI (mg/dl)	80-200	89.5	104.0	122.6	54.0^**#**^	52.8^**x**^	86.7	98.4	89.0	88.0	137.3
GOT (U/l)	20-250	131.0	81.0	82.2	83.0	90.4	65.0	90.0	133.0	67.0	123.3
GPT (U/l)	20-100	47.8	28.9	30.2	30.9	54.2	45.4	46.5	51.4	38.5	31.4
Glucose (mg/dl)	60-120	65.8	92.0	86.6	77.0	84.0	80.0	68.8	99.0	62.0	120.0
BUN (mg/dl)	10-40	28.3	10.0	20.2	22.0	22.0	23.8	23.0	22.5	23.0	22.3

CBC: complete blood count;CTR: control mice;ERW: mice treated with ERW;WBC: white blood cells; LYM: lymphocytes; GRAN: granulocytes; MID: mid-sized cells; HCT: hematocrit; MCV: mean corpuscular volume; RBC: red blood cells; HGB: hemoglobin; MCH: mean corpuscular hemoglobin; MCHC: mean corpuscular hemoglobin concentration; RDW: red cell distribution width; MPV: mean platelet volume; PLT: platelets; CHOL: cholesterol; TRI: triglycerides; GOT: serum Glutamic oxaloacetic transaminase; GPT: serum glutamic pyruvic transaminase; BUN: blood urea nitrogen (^x^p ≤ 0.05, *∗*p ≤ 0.01, ^#^p ≤ 0.001 compared with the control mice).

**Table 4:** Effects of ERW on apoptosis and cell cycle distribution of breast cancer cells after 72 hours of treatment.

	**Treatment**	**Sub-G1** ^†^		**G0/G1**		**S**		**G2/M**	
		**Mean ± SD**	**p-value**	**Mean ± SD**	**p-value**	**Mean ± SD**	**p-value**	**Mean ± SD**	**p-value**
**MCF-7**	CTR	3.08 ± 0.64		61.93 ± 1.81		12.66 ± 0.64		22.70 ± 1.80	
	Autoclaved ERW	3.58 ± 0.10		59.59 ± 0.97		13.91 ± 0.45		23.31 ± 1.42	
	ERW	19.60 ± 0.48	**<0.001** ^**1,2**^	49.34 ± 1.24	**<0.01** ^**1,2**^	11.88 ± 0.54		19.57 ± 1.28	

**MDA-MB-453**	CTR	5.16 ± 0.42		69.93 ± 0.11		10.36 ± 0.98		14.97 ± 1.30	
	Autoclaved ERW	5.61 ± 0.35		73.60 ± 2.63		9.16 ± 0.45		11.85 ± 1.81	
	ERW	16.83 ± 0.24	**<0.001** ^**1,2**^	61.18 ± 2.45	**<0.05** ^**1,2**^	8.18 ± 0.92		14.07 ± 1.86	

**TUBO**	CTR	6.65 ± 0.72		45.89 ± 2.95		15.13 ± 1.90		32.81 ± 0.32	
	Autoclaved ERW	6.04 ± 0.69		35.39 ± 0.57	**<0.01** ^**1**^	18.31 ± 0.89		40.75 ± 2.04	**<0.05** ^**1**^
	ERW	26.98 ± 3.95	**<0.01** ^**1,2**^	31.82 ± 0.28	**<0.01** ^**1**^	7.75 ± 1.79	**<0.05** ^**1,2**^	33.70 ± 2.51	**<0.05** ^**2**^

^†^Percentages of cells in the sub-G1, G0/G1, S, and G2/M phase were calculated using Cell Quest Pro 5.2 software. Results represent mean values±standard deviation (SD) from three independent experiments. The statistical significance of the effects obtained with ERW and autoclaved ERW was calculated *versus* those obtained with ^1^positive control medium (CTR) and ^2^autoclaved ERW.

## Data Availability

The data used to support the findings of this study are available from the corresponding author upon request.

## Supplementary Materials

Supplementary Materials
**Figure S1: effect of ERW on the expression and activation of ErbB receptor and prosurvival signaling pathway molecules and on apoptosis**. Western blotting analysis was performed on breast cancer cell lines that were treated with ERW and autoclaved ERW for 48 hours.
