# Supplementary material for: Electrochemically Reduced Water Delays Mammary Tumors Growth in Mice and Inhibits Breast Cancer Cells Survival In Vitro
Source: Evid Based Complement Alternat Med. 2018 Sep 26;2018:4753507. doi: 10.1155/2018/4753507 (PMC6196883; doi:10.1155/2018/4753507)
Supplement: Supplementary Materials — Figure S1: effect of ERW on the expression and activation of ErbB receptor and prosurvival signaling pathway molecules and on apoptosis. Western blotting analysis was performed on breast cancer cell lines that were treated with ERW and autoclaved ERW for 48 hours. [file 4753507.f1.pdf]

Supplementary data

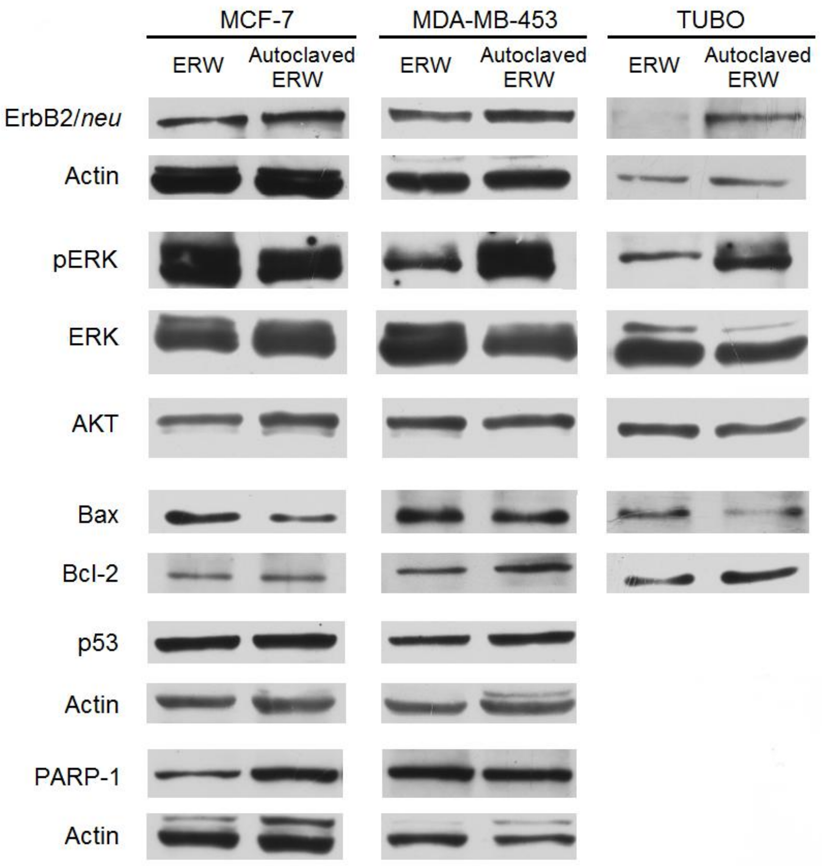

Fig. S1. Effect of ERW on the expression and activation of ErbB receptor and pro-survival signaling pathway molecules and on apoptosis. Western blotting analysis was performed on breast cancer cell lines that were treated with ERW and autoclaved ERW for 48 hours.
